# *Cucumber mosaic virus* infection as a potential selective pressure on *Arabidopsis thaliana* populations

**DOI:** 10.1371/journal.ppat.1007810

**Published:** 2019-05-28

**Authors:** Nuria Montes, Carlos Alonso-Blanco, Fernando García-Arenal

**Affiliations:** 1 Centro de Biotecnología y Genómica de Plantas (UPM-INIA), and E.T.S.I. Agronómica, Alimentaria y de Biosistemas, Campus de Montegancedo, Universidad Politécnica de Madrid, Pozuelo de Alarcón (Madrid), Spain; 2 Departamento de Genética Molecular de Plantas, Centro Nacional de Biotecnología, Consejo Superior de Investigaciones Científicas (CNB-CSIC), Campus Universidad Autónoma, Cantoblanco, Madrid, Spain; Institute of Microbiology, CHINA

## Abstract

It has been proposed that in wild ecosystems viruses are often plant mutualists, whereas agroecosystems favour pathogenicity. We seek evidence for virus pathogenicity in wild ecosystems through the analysis of plant-virus coevolution, which requires a negative effect of infection on the host fitness. We focus on the interaction between *Arabidopsis thaliana* and *Cucumber mosaic virus* (CMV), which is significant in nature. We studied the genetic diversity of *A*. *thaliana* for two defence traits, resistance and tolerance, to CMV. A set of 185 individuals collected in 76 *A*. *thaliana* Iberian wild populations were inoculated with different CMV strains. Resistance was estimated from the level of virus multiplication in infected plants, and tolerance from the effect of infection on host progeny production. Resistance and tolerance to CMV showed substantial genetic variation within and between host populations, and depended on the virus x host genotype interaction, two conditions for coevolution. Resistance and tolerance were co-occurring independent traits that have evolved independently from related life-history traits involved in adaptation to climate. The comparison of the genetic structure for resistance and tolerance with that for neutral traits (*Q*_*ST*_/*F*_*ST*_ analyses) indicated that both defence traits are likely under uniform selection. These results strongly suggest that CMV infection selects for defence on *A*. *thaliana* populations, and support plant-virus coevolution. Thus, we propose that CMV infection reduces host fitness under the field conditions of the wild *A*. *thaliana* populations studied.

## Author summary

Plant-virus coevolution has not been demonstrated in any wild system, and it has been proposed that viruses often would be mutualistic symbionts, rather than pathogens, in wild plant ecosystems. We analyse if viruses are virulent pathogens of plants in wild ecosystems and, consequently, plants have evolved defences against virus infection. To test this hypothesis, we studied the genetic diversity of *Arabidopsis thaliana* for two defence traits, resistance and tolerance, to *Cucumber mosaic virus* (CMV) at a regional scale in the Iberian Peninsula. Resistance and tolerance to CMV showed substantial genetic variation within and between host populations, and depended on the virus x host genotype interaction, two conditions for coevolution. Resistance and tolerance were independent traits that co-occurred at the population and regional scales, and that have evolved independently from other adaptive life-history traits. Analyses also indicated that resistance and tolerance are likely under selection, most likely due to virus infection. These results support a hypothesis of plant-virus coevolution and contribute to demonstrate that plant viruses may be virulent parasites of plants in wild ecosystems.

## Introduction

It is commonly accepted that hosts and pathogens coevolve [1]. This concept rests on the assumption that pathogens are virulent parasites, defining virulence as the negative impact of infection on the host fitness. As a consequence, hosts will evolve defences to limit pathogen infection, or to compensate for its costs [2]. In plants, the two major defences against pathogens are resistance, defined as the ability of the host to limit infection and/or parasite multiplication and tolerance, which limits the fitness effect of a given parasite burden, i.e., specifically decreases virulence [3,4]. As host defences may reduce the parasite’s fitness, hosts and parasites may coevolve, coevolution being the process of reciprocally adaptive genetic change in two or more species [1]. Evidence for host pathogen coevolution is not abundant. For plants it derives mostly from studies of agroecosystems in which the pathogen evolves in response to the deployment of resistance in the host population [5]. These studies have provided the bases for theory on host-pathogen coevolution, including the gene-for-gene model of host-pathogen interaction [6,7]. However, coevolution requires certain conditions to be met [1]: i) genetic variation in the relevant host (e.g., resistance, tolerance) and pathogen (e.g., infectivity, virulence) traits; ii) reciprocal effects of the relevant traits of the interaction on the fitness of host and pathogen; iii) dependence of the outcome of the host-pathogen interaction on the combination of host and pathogen genotypes involved. These conditions must be analysed in wild systems, in which the host may evolve in response to environmental pressures, including pathogen infection, at odds with agricultural systems. Evidence for plant-pathogen coevolution from wild pathosystems is limited to a few instances, all involving fungal, oomycete or bacterial pathogens [8,9].

To our knowledge, plant-virus coevolution has not been demonstrated in any wild system. In fact, it has been proposed that viruses often would be mutualistic symbionts, rather than pathogens, in wild plant ecosystems [10–12], and it has been shown that virus infection may be detrimental or positive for the host depending on the environment [13,14]. Hence the interest in seeking evidence about whether viruses are plant pathogens in wild ecosystems and viruses and plants co-evolve, or if virus virulence is the result of the specific conditions of agroecosystems. Reports of negative effects of virus infection in wild plants in their natural habitats are not abundant [e.g., [15–22] and indicate that effects may largely depend on site or host population [23], but the genetic variation of defence and virulence has not been analysed in these systems.

To analyse plant-virus coevolution in wild ecosystems we have chosen the system *Arabidopsis thaliana* L. Heynh. (Brassicaceae)-*Cucumber mosaic virus* (*Cucumovirus*, *Bromoviridae*), (CMV). *A*. *thaliana* is an annual semelparous species with two distinct developmental periods: the vegetative growth period, producing a rosette of leaves, and the reproductive period in which the inflorescence grows, new flowers are produced continuously, and older flowers develop into siliques [24]. It is a cosmopolitan species, with a broad native distribution in Eurasia and Africa [25–27]. The Iberian Peninsula has been shown to contain the largest *A*. *thaliana* diversity in Eurasia due to its colonization from different refugia after the last glaciations [26–29]. In Iberia, *A*. *thaliana* occurs in a variety of habitats, and substantial genetic variation has been described, within and among populations, for relevant adaptive traits including phenological traits like flowering time and seed dormancy [30–33]. Although wild populations of *A*. *thaliana* have been shown to contain ample genetic and phenotypic diversity for responses to herbivores and pathogens [34–38], the diversity for traits related to plant-virus interactions has not been systematically analysed.

CMV is an RNA virus with the broadest host range including about 1,200 species in more than 100 plant families. CMV is horizontally transmitted by many species of aphids in a non-persistent manner, and through the seed with efficiencies that depend on the genotypes of CMV and the plant species [39]. In *A*. *thaliana*, seed transmission rates vary between 2 and 8% [40, 41]. CMV isolates are highly diverse and have been classified into subgroups IA, IB and II, based on the nucleotide sequence similarity of their genomic RNAs [39,42].

Analyses of the incidence of five viruses in six wild *A*. *thaliana* populations from central Spain during 10 years showed that CMV was most prevalent, up to 80% according to population and year [43,44], indicating that the *A*. *thaliana*–CMV interaction is significant in nature. As in other hosts monitored in the Iberian Peninsula, Subgroup IA isolates are most prevalent [44–46]. Our group has analysed the role of resistance and tolerance in this interaction. The infection of 21 wild genotypes of *A*. *thaliana* representing the variation of the species in Eurasia with three CMV strains, showed that quantitative resistance to CMV depended on the interaction between host genotype x virus strain, and was a host trait with moderate to high heritability [47]. Virulence, estimated as the effect of infection on viable seed production, did not correlate with virus load, due to host genotype x virus strain-specific tolerance and, again, tolerance was a host trait with moderate to high heritability [47]. Interestingly, tolerance was positively correlated with the length of post-embryonic development (life span) of the host genotypes [47], and was due, at least in part, to host life-history trait modification upon infection: long life span genotypes delayed flowering upon infection and re-allocated resources from vegetative growth to reproduction, thus decreasing the effects of infection on progeny production, i.e., attaining tolerance [48]. It remains to be shown that defence polymorphisms result from the selection applied by CMV infection, and not by any other environmental factor known to modulate plant developmental architecture and phenology, such as life span, flowering time and plant size, which are known to have a role in adaptation of *A*. *thaliana* to the abiotic environment [49–51].

In this work we study the genetic diversity of *A*. *thaliana* for resistance and tolerance to CMV at a regional scale in the Iberian Peninsula. To this end, we exploit a collection of 76 natural populations covering the wide ecological, environmental and genetic diversities of *A*. *thaliana* in this region [32,49]. We address the following questions: i) Which is the amount of genetic diversity for resistance and tolerance to different CMV genotypes? ii) Are the geographic and environmental climatic patterns of resistance and tolerance similar or different from those of related adaptive life history traits of *A*. *thaliana*? iii) Are resistance and tolerance traits under natural selection? Addressing these questions is crucial to determine if CMV infection has a negative impact on its host fitness under natural field conditions and, consequently, if there is coevolution between *A*. *thaliana* and CMV.

## Results

### Genetic variation for resistance and tolerance to CMV in *A*. *thaliana*

**Fig 1:**
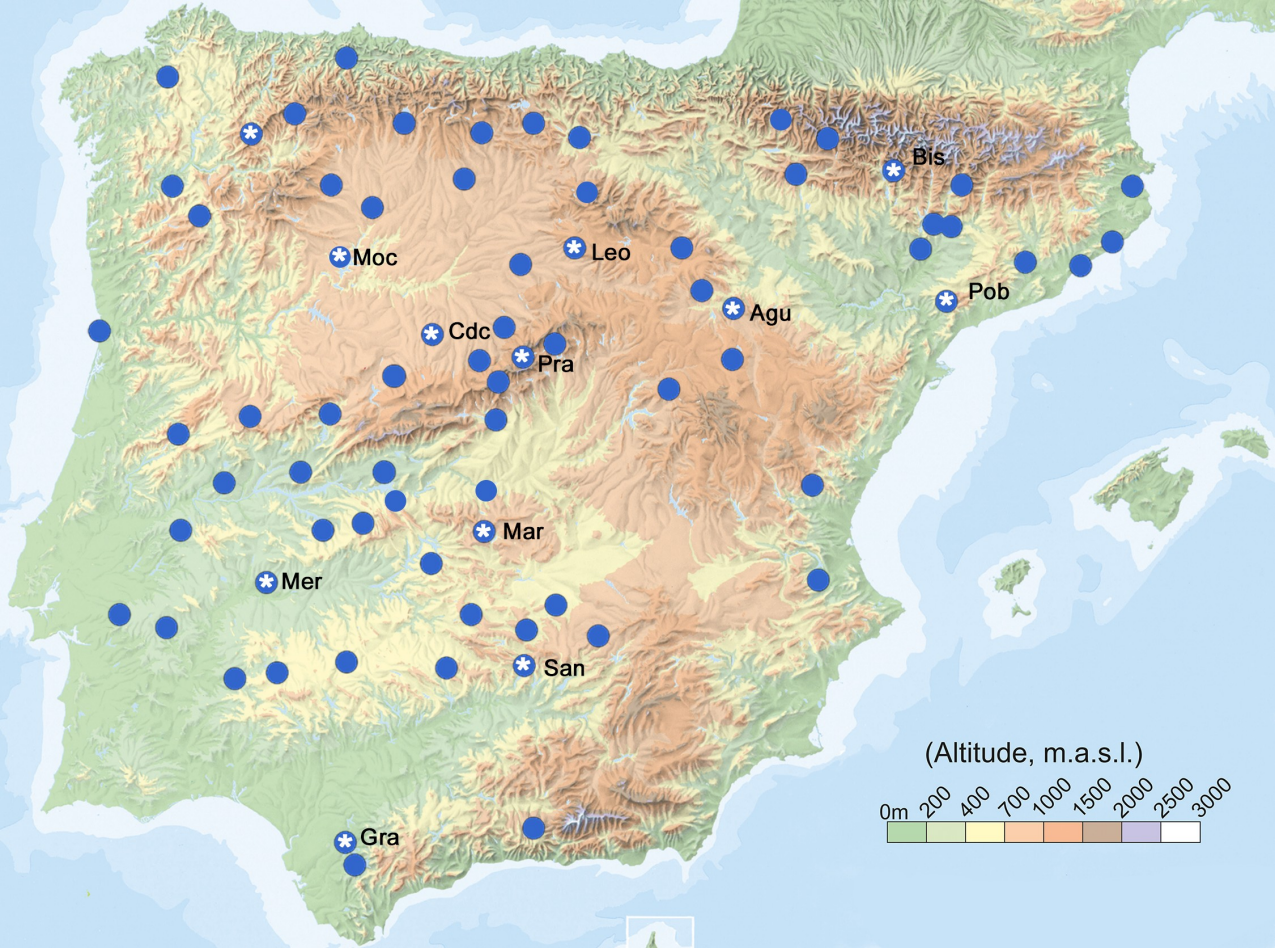
Geographic distribution of *Arabidopsis thaliana* populations analysed in this study. Circles indicate population locations. Circles with asterisk indicate the 12 populations used for within/between population analyses, their names appearing next to them.

To estimate the genetic diversity for resistance and tolerance to CMV, 76 *A*. *thaliana* wild genotypes collected in different natural populations from the Iberian Peninsula (Fig 1 and S1 Table) were assayed. We consider here a population as the set of *A. thaliana* plants growing in a specific geographical site. Plants were inoculated after eight-week vernalisation, using two CMV isolates from Iberian *A*. *thaliana* populations, Cdc-CMV and Lro-CMV. All 76 genotypes were systemically infected by both CMV isolates, no immunity or hypersensitive resistance reaction being detected.

**Fig 2:**
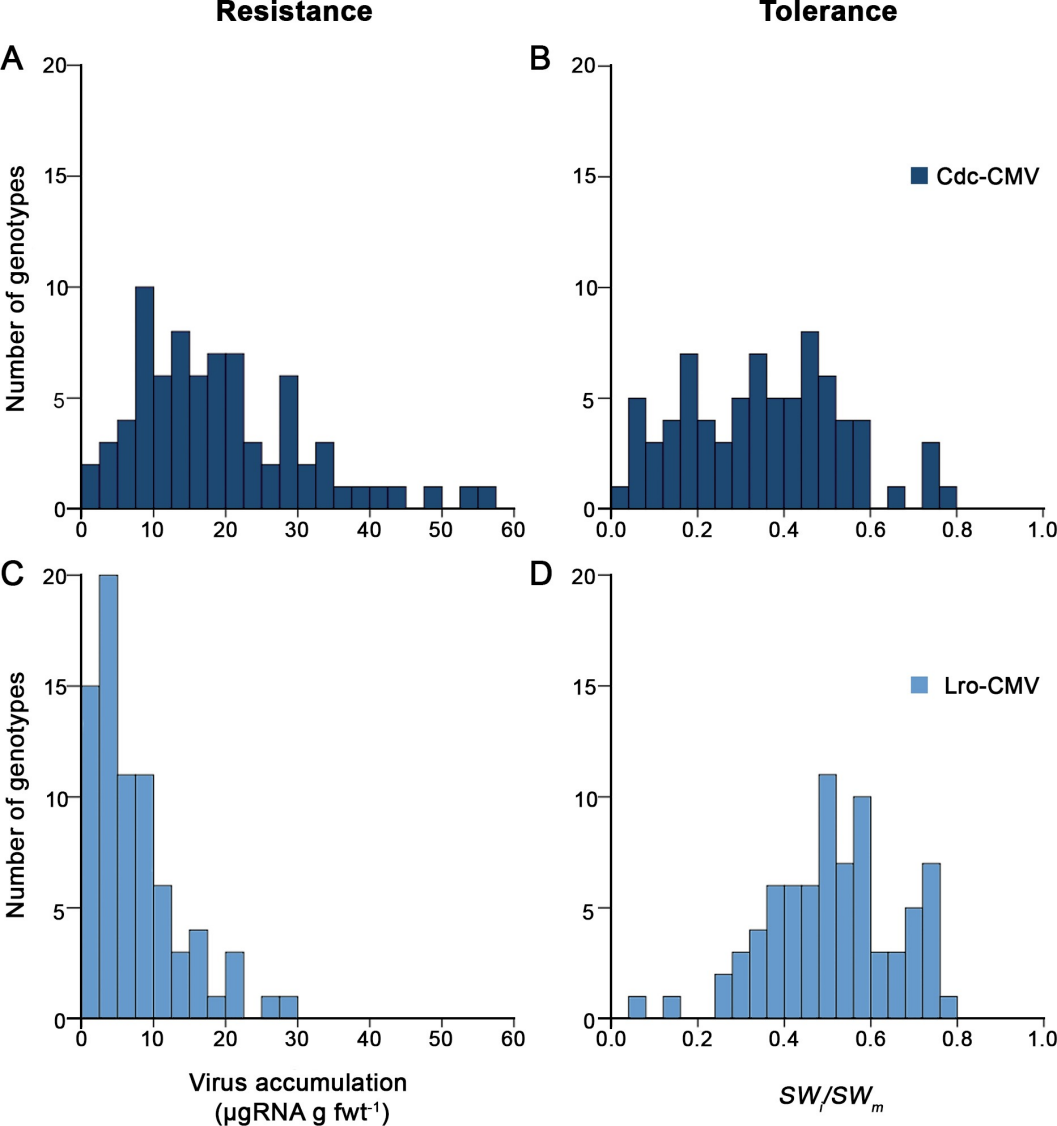
Variation for resistance and tolerance to CMV in *A*. *thaliana*. Frequency distributions are for accumulation (μg RNA g fresh leaf weight^-1^) of (A) Cdc-CMV and (B) Lro-CMV RNA, and of the effect of infection by (C) Cdc-CMV and (D) to Lro-CMV on seed production.

Resistance was estimated from the levels of virus multiplication, quantified as virus RNA accumulation. RNA accumulation varied between 0.26 and 56.28 μg of virus RNA g fwt^-1^ (Table 1, Fig 2 and S2 and S3 Tables), variation significantly depending on the *A*. *thaliana* genotype (*F*_*75*,*816*_ *=* 2.98, *P*<10^−4^), the virus isolate (*F*_*1*,*816*_ *=* 113.30, *P*<10^−4^) and the interaction virus isolate x host genotype (*F*_*75*,*816*_ *=* 75.82, *P*<10^−4^), which together explained 88% of the variance (24.6% for *A*. *thaliana* genotype, 38.9% for virus isolate and 24.5% for their interaction). On average, Cdc-CMV accumulated to higher levels than Lro-CMV (19.28±1.37 and 7.53±0.71 μg virus RNA g fwt^-1^, respectively) (Fig 2 and Table 1). Virus accumulation in the host plant showed high heritability for both CMV isolates, with an average of 0.8 (Table 1), heritability being defined as the genetic component of the variance of the trait.

**Table 1:** Values (mean and range of variation), heritability and autocorrelation, of life history and defence traits to CMV in the Iberian population of *A*. *thaliana*.

					Autocorrelation	
Trait^a^	*n*^*b*^	Mean±SE^c^	Min-Max^d^	*h*^*2*^_*b*_ ^*e*^	*Moran's I* ^f^	Distance^g^
*RW*	76	0.50±0.04	0.04–1.80	0.96	0.43–0.85	219.31
*IW*	76	1.83±0.06	0.66–3.48	0.71	0.41–0.89	204.69
*SW*	76	0.85±0.02	0.31–1.23	0.61	0.23–0.33	153.47
*GP*	76	117.72±0.80	103.40–140.71	0.92	0.43–0.59	191.75
*LP*	76	184.26±0.76	170.80–198.00	0.72	ns	ns
Resistance to Cdc-CMV^h^	76	19.28±1.37	0.56–56.28	0.81	ns	ns
Resistance to Lro-CMV^h^	76	7.53±0.71	0.26–27.83	0.78	ns	ns
Tolerance to Cdc-CMV^i^	76	0.35±0.02	0.03–0.77	0.70	ns	ns
Tolerance to Lro-CMV^i^	76	0.51±0.02	0.06–0.79	0.54	ns	ns

^a:^ Traits are: Rosette weight (*RW*), inflorescence without seed weight (*IW*) and viable seed weight (*SW*), expressed in g, and growth period (*GP*) and life span (*LP*), expressed in days.

^b:^ number of individuals.

^c:^ Mean value and standard error of at least 5 replicated plants.

^d:^ Minimum and maximum values of the trait.

^e:^ Broad sense heritability expressed as percentage of genetic variation.

^f:^ Values of significant values of *Moran's index* (*P*<0.001).

^g:^ Geographic distance in km, showing significant values of Moran's index.

^h:^ Resistance is expressed as virus accumulation (μg of virus RNA g fresh leaf weight^-1^).

^i:^ Tolerance is expressed as effect of infection on viable seed production (*SW*_*i*_*/SW*_*m*_).

ns: non-significant.

Tolerance was estimated from the effect of virus infection on progeny production. Since CMV infection does not affect seed viability or the weight of single seeds in a large number of *A*. *thaliana* genotypes [14,47,52], tolerance was estimated as the ratio of seed weight in infected to mock-inoculated plants (*SW*_*i*_*/SW*_*m*_), which varied between 0.03 and 0.79 (Table 1, Fig 2, S2 and S3 Tables). As for resistance, tolerance significantly depended on *A*. *thaliana* genotype (*F*_*76*,*816*_ *=* 3.10, *P*<10^−4^), virus isolate (*F*_*1*,*816*_ *=* 71.40, *P*<10^−4^) and their interaction (*F*_*75*,*816*_ *=* 5.18, *P*<10^−4^), which together explained 73% of the variance (26.9% for *A*. *thaliana* genotype, 25.0% for virus isolate and 21.2% for their interaction). Tolerance to Cdc-CMV (0.35±0.02) was lower than tolerance to Lro-CMV (0.51±0.02) (Fig 2 and Table 1). Tolerance in the host showed medium to high heritability, between 0.70 and 0.54 for Cdc-CMV and Lro-CMV, respectively.

No significant relationship was detected between virus RNA accumulation and *SW*_*i*_*/SW*_*m*_ across genotypes nor within genotypes (*r*≤-0.11, *P*≥0.358). Together, results show that natural populations of *A*. *thaliana* contain substantial but independent genetic variation for resistance and tolerance to CMV.

### Geographic and climatic patterns of *A*. *thaliana* resistance and tolerance to CMV

To determine if there is a geographic pattern for the genetic diversity for CMV resistance or tolerance, we first analysed the spatial autocorrelation of both variables. Neither virus accumulation nor *SW*_*i*_*/SW*_*m*_ showed significant spatial autocorrelation at any geographic scale (*P*>0.050) (Table 1).

Second, to find if these defence traits might be associated with the climate, we analysed their relationship with climatic variables from the original local populations. Neither the accumulation of any of the two CMV isolates, nor the *SW*_*i*_*/SW*_*m*_ ratio in plants infected by any of them, significantly correlated with any of the analysed abiotic variables (see Methods and S4 Table) according to Dutilleul’s t-tests (*r*≤-0.30, *P*≥0.039), SARs (*F*≤7.185, *P*≥0.009), or Mantel tests (*r*≤-0.12, *P*≥0.019) (S4 Table).

Finally, we analysed if there is a relationship between the genetic diversity for CMV defence traits and the overall genetic diversity of the *A*. *thaliana* wild genotypes estimated from neutral markers (250 SNPs). Mantel tests between pair-wise genetic distances estimated from neutral markers and pair-wise differences for virus RNA accumulation or *SW*_*i*_*/SW*_*m*_ did not detect any significant correlation in relation to Cdc-CMV or Lro-CMV (*r*≤-0.04, *P*≥0.403).

### Genetic diversity and geographic or climatic patterns of life history traits in *A*. *thaliana*

Tolerance to CMV in *A*. *thaliana* is related to the host life history traits, as tolerance is due in part to a reallocation of resources from growth to reproduction, which depends on the allometry of the vegetative to reproduction organs, (*SW+IW)/RW* [48]. In the 76 wild genotypes, resource reallocation upon infection by both CMV isolates also depended on plant allometry, being more efficient in genotypes with lower (*SW+IW)/RW (F*_*1*,*76*_>14.58, *P*<10^−4^), and the effect of infection by both CMV isolates on *RW* correlated positively with *LP* and *RW* of mock-inoculated plants (*r*≤0.29, *P*≥0.022). Neither resistance nor tolerance correlated with viable seed production of mock-inoculated controls (*r*≤0.02, *P*≥0.540).

We then analysed several life history traits related with growth and phenology in eight-week vernalised mock-inoculated plants of the 76 wild genotypes. Rosette weight (*RW*), inflorescence without seeds weight (*IW*) and seed weight (*SW*), growth period (*GP*) and life-span (*LP*) significantly differed between genotypes (*F*_75,399_≥9.23, *P*<10^−4^) (S2 and S3 Tables). Heritability of these traits was high, between 0.61 and 0.96 (Table 1). Overall, *A*. *thaliana* populations display a large genetic variation for the analysed life history traits, as in previous works [33,49,50].

**Fig 3:**
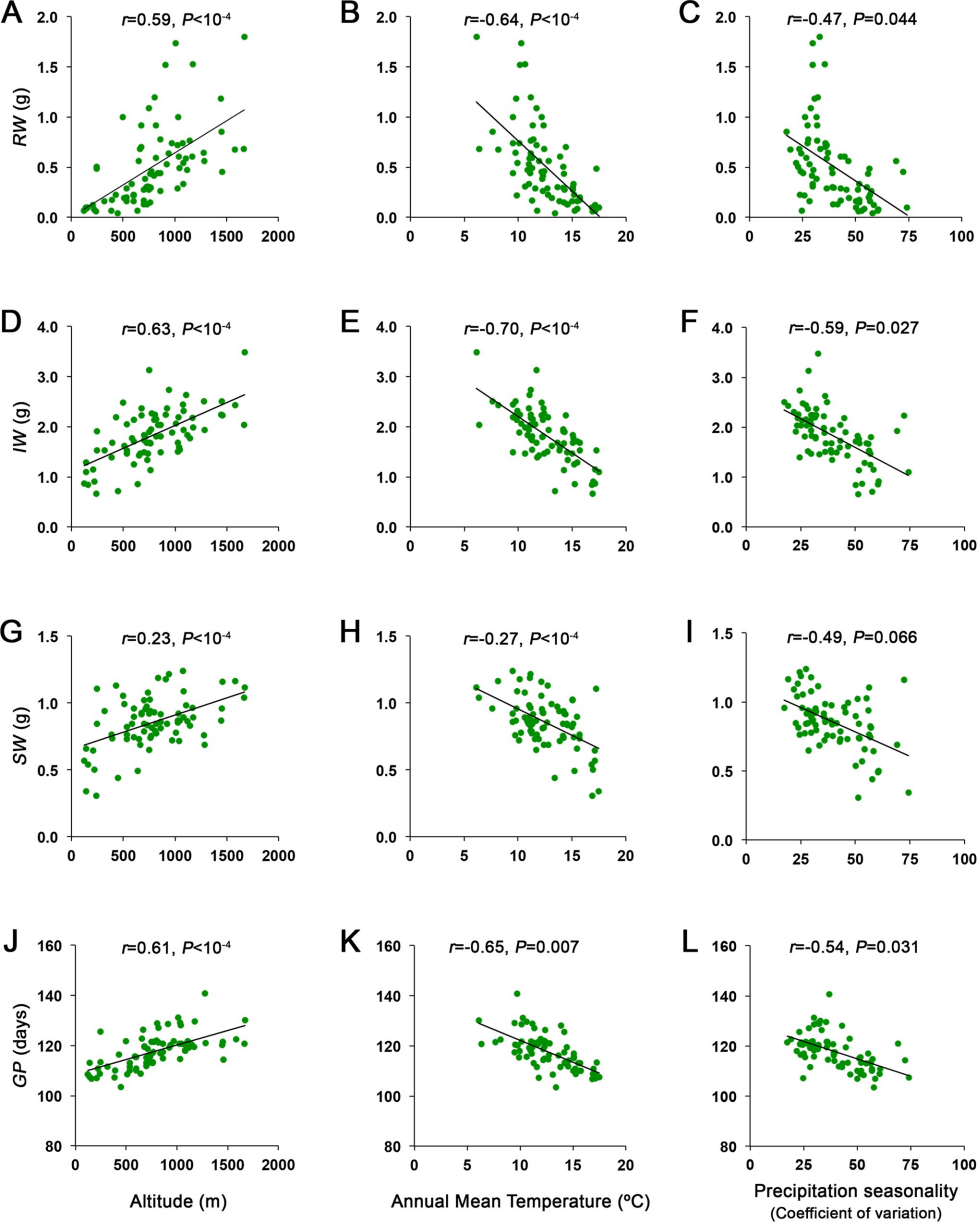
Relationships between life-history traits and geographic or climatic factors. Correlations are shown *(*A, B, C*)* for rosette weight (*RW)*, (D, E, F) inflorescence weight (*IW)*, (G, H, I) seed weight (*SW)* and (J, K, L) growth period (*GP)* with altitude, annual mean temperature and precipitation seasonality. Values are means of at least five replicates per plant genotype.

Rosette weight (*RW*), inflorescence without seeds weight (*IW*) and seed weight (*SW*) significantly differed between genotypes (*F*_75,399_≥9.23, *P*<10^−4^) (S2 Table). To determine if life history traits related to tolerance showed similar or different geographic patterns than CMV defences, we analysed their autocorrelation. All growth and phenological traits showed significant spatial autocorrelation up to 153 km (Table 1). Furthermore, we analysed the relationship between these life history traits and climate using Dutilleul’s t-test, univariate SAR models and Mantel tests (S4 Table). Overall, *RW*, *IW*, *SW* and *GP*, but not *LP*, were positively correlated with altitude and negatively correlated with most climatic variables, including annual mean, minimal and maximal temperature, and precipitation seasonality (Fig 3 and S4 Table). These analyses showed *A*. *thaliana* genotypes from higher altitude, lower temperatures and higher precipitation seasonality flowered later, developed larger rosettes and inflorescences and produced more seeds. Moreover, Mantel tests showed that genetic distance was positively correlated with *IW*, *SW*, *GP* (*r*>0.15, *P*<0.008) and marginally with *RW* (*r* = 0.08, *P* = 0.078) but not with *LP* (*r*<-0.02, *P*>0.700).

In contrast to CMV defence traits, life history traits related with resource allocation vary according to the climatic environment where populations evolved. Therefore, resistance and tolerance to CMV show different evolutionary histories than life history traits, likely reflecting distinct abiotic and biotic environmental selective forces acting on each group of traits.

### *A*. *thaliana* population differentiation for resistance and tolerance to CMV

To quantify the distribution of genetic diversity for CMV defence traits within and among *A*. *thaliana* populations we analysed ten randomly sampled individual plants (henceforth named as “individuals”) from 10 or 12 Iberian populations, which were tested for their resistance and tolerance to two CMV isolates. One isolate from an Iberian population of *A*. *thaliana* (Cdc-CMV) and a reference isolate (Fny-CMV) were chosen because they had been used in previous work [43,47,48]. Since CMV resistance and tolerance depend on the environment [14,48,53], two experiments were performed with different vernalisation period lengths, as vernalisation affects life history traits relevant to tolerance such as rosette size, rosette leaf number, flowering time [33,49,54], and seed germination [55]. An eight-week vernalisation treatment simulated a cold winter, whereas a four-week vernalisation simulated a mild winter, as often occur across years in the original population locations. In both experiments, all individuals were systemically infected by both CMV isolates. The two experiments yielded similar results. For clarity only results of the long vernalisation treatment experiment are presented in the text, but results of the short-vernalisation are shown in S5 Table.

**Fig 4:**
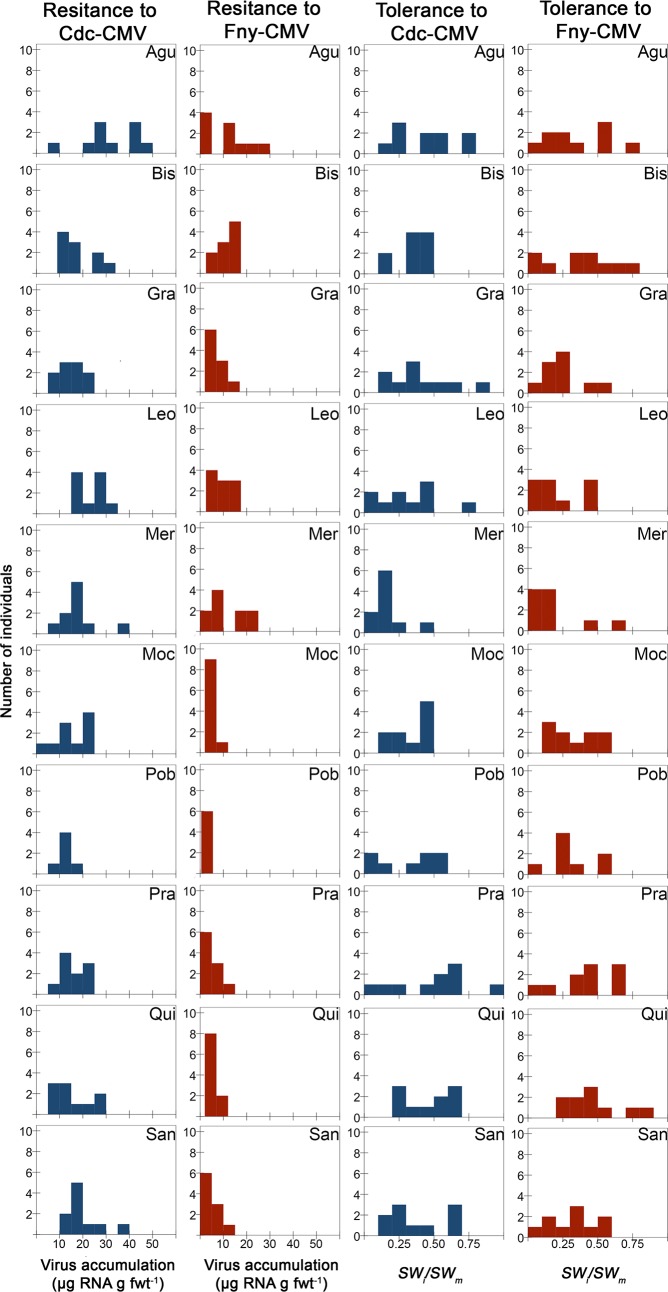
Variation for resistance and tolerance to CMV within and among wild *A*. *thaliana* populations. Frequency distributions of resistance (virus accumulation, μg RNA g fresh leaf weight^-1^) and tolerance (*SW*_*i*_*/SW*_*m*_) to Cdc-CMV (blue) and Fny-CMV (red). Three-letter code for each population is as in Fig 1.

Virus accumulation varied considerably among individuals within populations (Fig 4, Table 2 and S6 Table). The average virus accumulation in each population ranged from 2.62 to 32.02 μg virus RNA g fwt^-1^. The heritability of virus accumulation varied between 0.23 and 0.90 depending on CMV isolate and host population (Table 2). Virus accumulation significantly depended on the virus isolate (*F*_1,884_ = 67.88, *P*<10^4^), the *A*. *thaliana* population (*F*_9,884_ = 2.40, *P* = 0.059) (Fig 4 and Table 2), the *A*. *thaliana* individual nested to population (*F*_88,884_ = 2.54, *P*<10^-4^), and on the interactions CMV isolate x *A*. *thaliana* population (*F*_9,884_ = 4.11, *P*<10^-4^) and CMV isolate x *A*. *thaliana* individual nested to population (*F*_88,884_ = 7.08, *P*<10^-4^). CMV isolate, *A*. *thaliana* individual nested to population and their interaction explained 49.7, 3.3 and 14.6% of the variance, respectively, while *A*. *thaliana* population and the interaction CMV isolate x population explained 7.0 and 5.5%. The average accumulation was higher for Cdc-CMV than for Fny-CMV (18.61±2.76 and 7.37±1.74 μg virus RNA g fwt^-1^, respectively). Besides, average values of Cdc-CMV accumulation over individuals and populations correlated significantly, or marginally, with the corresponding values of Fny-CMV accumulation (*r*_*s*_ = 0.32, *P* = 0.001; *r*_*s*_ = 0.60, *P* = 0.067, respectively), indicating that, in general, individuals and populations that were more resistant to Cdc-CMV were also more resistant to Fny-CMV.

**Table 2:** Values (mean and range of variation) and heritability of defence traits to CMV in Iberian populations of *A*. *thaliana*^a^.

Population		Resistance to Cdc-CMV^b^	Resistance to Fny-CMV^b^	Tolerance to Cdc-CMV^c^	Tolerance to Fny-CMV^c^
**Agu**	Mean±SE^d^	32.02±3.64	11.85±2.84	0.43±0.07	0.36±0.07
	Min-Max^e^	7.98–45.64	2.16–26.11	0.14–0.78	0.08–0.74
	n^f^	10	10	10	10
	***h***^***2***^_***b***_ ^***g***^	0.74	0.90	0.49	0.44
**Bis**	Mean±SE^d^	17.05±2.31	10.91±1.11	0.34±0.04	0.37±0.07
	Min-Max^e^	9.37–30.04	3.31–14.14	0.12–0.48	0.03–0.71
	n^f^	10	10	10	10
	***h***^***2***^_***b***_ ^***g***^	0.74	0.58	0.38	0.8
**Gra**	Mean±SE^d^	15.77±1.68	6.24±1.15	0.40±0.07	0.26±0.05
	Min-Max^e^	6.69–24.45	2.42–13.97	0.12–0.84	0.04–0.54
	n^f^	10	10	10	10
	***h***^***2***^_***b***_ ^***g***^	0.66	0.68	0.64	0.741
**Leo**	Mean±SE^d^	22.36±1.90	9.48±1.40	0.32±0.07	0.22±0.05
	Min-Max^e^	15.45–32.05	3.59–16.29	0.05–0.74	0.03–0.46
	n^f^	10	10	10	10
	***h***^***2***^_***b***_ ^***g***^	0.62	0.79	0.61	0.77
**Mer**	Mean±SE^d^	17.58±2.56	11.55±2.35	0.16±0.04	0.19±0.06
	Min-Max^e^	5.37–36.76	3.89–24.15	0.04–0.46	0.05–0.67
	n^f^	10	10	10	10
	***h***^***2***^_***b***_ ^***g***^	0.79	0.89	0.32	0.67
**Moc**	Mean±SE^d^	15.49±2.25	5.15±0.43	0.35±0.04	0.32±0.05
	Min-Max^e^	4.08–24.43	2.92–7.07	0.13–0.49	0.12–0.57
	n^f^	10	10	10	10
	***h***^***2***^_***b***_ ^***g***^	0.71	0.34	0.27	0.47
**Pob**	Mean±SE^d^	14.91±1.71	2.62±0.60	0.36±0.07	0.33±0.05
	Min-Max^e^	5.33–22.55	0.94–7.01	0.02–0.59	0.07–0.57
	n^f^	9	9	9	9
	***h***^***2***^_***b***_ ^***g***^	0.52	0.23	0.74	0.10
**Pra**	Mean±SE^d^	15.04±2.15	4.78±0.93	0.47±0.09	0.41±0.07
	Min-Max^e^	5.19–24.98	1.99–10.26	0.06–0.93	0.04–0.68
	n^f^	9	9	9	9
	***h***^***2***^_***b***_ ^***g***^	0.635	0.63	0.55	0.66
**Qui**	Mean±SE^d^	15.23±2.34	6.21±0.78	0.47±0.06	0.46±0.06
	Min-Max^e^	6.26–27.61	3.87–11.85	0.20–0.69	0.24–0.81
	n^f^	10	10	10	10
	***h***^***2***^_***b***_ ^***g***^	0.68	0.34	0.64	0.52
**San**	Mean±SE^d^	20.00±2.48	4.16±1.46	0.36±0.06	0.32±0.05
	Min-Max^e^	11.38–38.89	0.08–12.18	0.11–0.66	0.10–0.57
	n^f^	10	10	10	10
	***h***^***2***^_***b***_ ^***g***^	0.69	0.81	0.47	0.32
**Average of populations**	Mean±SE	18.53±1.65	7.25±0.30	0.37±1.09	0.31±0.02
	Min-Max	14.91–32.02	2.62–11.85	0.16–0.47	0.22–0.46
** **	***h***^***2***^_***b***_^***g***^	0.60	0.62	0.51	0.55
**Over populations**	***h***^***2***^_***b***_^***g***^	0.77	0.85	0.57	0.55

^a:^ Values are for plants inoculated after an eight-week vernalisation period.

^b:^ Resistance is expressed as virus accumulation (μg of virus RNA g fresh leaf weight^-1^).

^c:^ Tolerance is expressed as effect of infection on viable seed production (*SW*_*i*_*/SW*_*m*_).

^d:^ Mean ± SE: Mean value and standard error (SE) of at least 5 replicated plants.

^e:^ Minimum and maximum values of the trait.

^f:^ number of individuals

^g:^ Broad sense heritability expressed as percentage of genetic variation.

CMV tolerance also showed substantial variation among individuals within populations, *SW*_*i*_*/SW*_*m*_ values ranging between 0.02 and 0.93 for Cdc-CMV, and between 0.03 and 0.81, for Fny-CMV-infected plants (Fig 4, Table 2 and S6 Table). Average *SW*_*i*_*/SW*_*m*_ values in each population varied from 0.16 to 0.47 (Fig 4 and Table 2), indicating a lower range of variation among than within populations. Heritability of tolerance varied between 0.10 and 0.80 depending on CMV isolate and host population (Table 2). *SW*_*i*_*/SW*_*m*_ varied significantly depending on virus isolate (*F*_1,884_ = 5.30, *P* = 0.046), *A*. *thaliana* population (*F*_9,884_ = 2.40, *P* = 0.059) (Fig 4 and Table 2), *A*. *thaliana* individual nested within population (*F*_88,884_ = 2.445, *P* = 0.023) and the interaction CMV isolate x *A*. *thaliana* individual (*F*_88,884_ = 2.65, *P*<10^−4^). *A*. *thaliana* individual and the interaction CMV isolate x *A*. *thaliana* individual explained a larger proportion of *SW*_*i*_*/SW*_*m*_ variance (32.2%, 16.1%, respectively) than CMV isolate or *A*. *thaliana* population (1.6%, 6.4%, respectively). When averaged over all individuals, tolerance to Cdc-CMV (0.36±0.06) and Fny-CMV (0.32±0.06) were similar. Average values of *SW*_*i*_*/SW*_*m*_ in Cdc-CMV-infected plants correlated across individuals and populations with those of Fny-CMV-infected plants (*r*_*s*_ = 0.73, *P* = 0.016; *r*_*s*_ = 0.60, *P*<10^−4^, respectively). Thus, as for resistance, the individuals and populations more tolerant to Cdc-CMV were also, in general, more tolerant to Fny-CMV. However, values of virus accumulation and *SW*_*i*_*/SW*_*m*_ did not correlate over individuals for any CMV isolate (*r*_*s*_≤0.09, *P*≥0.129). Together, these results indicate that *A*. *thaliana* defences against CMV infection depend on the host genetic variation determining the specific defences.

### Comparison of genetic differentiation among *A*. *thaliana* populations between quantitative traits and neutrals markers

**Fig 5:**
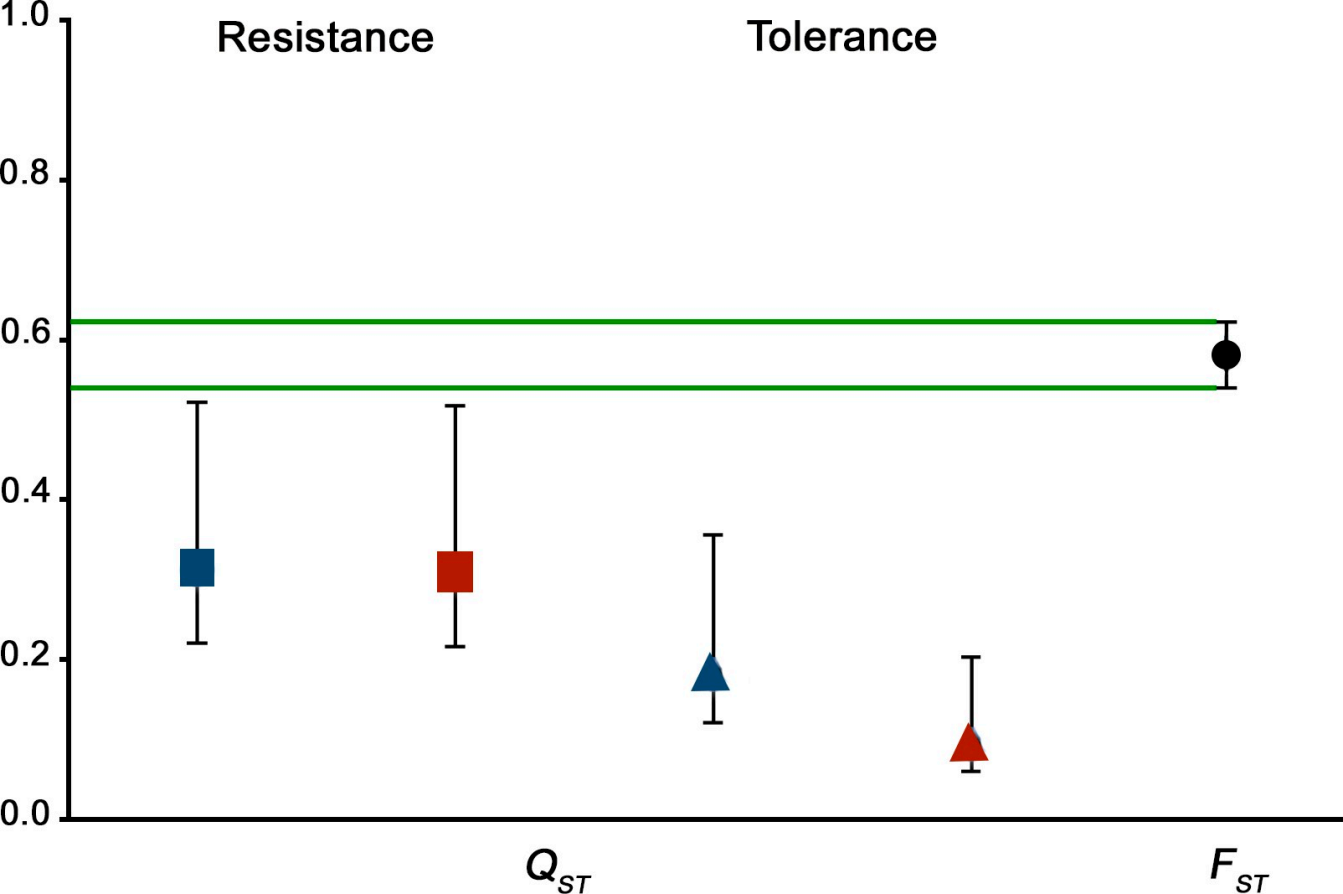
Genetic differentiation among wild *A*. *thaliana* populations for CMV resistance and tolerance traits, or for neutral markers. Fig shows values of quantitative genetic differentiation (*Q*_*ST*_) for resistance (squares) and tolerance (triangles), to Cdc-CMV (blue) or Fny-CMV (red), and for neutral genetic differentiation (*F*_*ST*_) (black circle), among ten *A*. *thaliana* populations. 95%CI are indicated.

To find out if CMV defence traits of *A*. *thaliana* might be under selection, we compared the genetic differentiation among populations for CMV resistance and tolerance, with that for neutral genetic variation (Fig 5). Two-hundred and fifty genome-wide SNPs, distinguished 74 different genotypes among the 120 individuals analysed (S7 Table) and were used to calculate *F*_*ST*_ values. As in the previous section, analyses are based on the results of the long vernalisation treatment experiment, analyses based on the short-vernalisation treatments gave similar results and are shown in S5 Table.

The estimated average genetic differentiation among populations for neutral markers was 0.58 (95%CI = 0.54–0.62), which is presumed to reflect the demographic history of the populations. Genetic differentiation among populations for quantitative traits was estimated by *Q*_*ST*_ values, leading to a similar average value of 0.31 (95%CI = 0.22–0.52) for accumulation of both CMV isolates. The average *Q*_*ST*_ estimated for *SW*_*i*_*/SW*_*m*_ was 0.18 (95%CI = 0.12–0.35) for Cdc-CMV and 0.10 (95%CI = 0.06–0.21) for Fny-CMV-infected plants. Therefore, the genetic variation of *A*. *thaliana* for both defence traits is distributed mostly within populations. *Q*_*ST*_ values for resistance and tolerance to Cdc-CMV and Fny-CMV were significantly smaller than *F*_*ST*_ values (Fig 5), thus indicating that *A*. *thaliana* populations are genetically less differentiated for resistance and tolerance to two CMV isolates than for neutral markers.

Furthermore, we analysed if *F*_*ST*_ and *Q*_*ST*_ values followed a pattern of isolation-by-distance. Mantel tests detected a significant correlation between *F*_*ST*_ values and geographic distance between pairs of populations (*r* = 0.50, *P*<10^−4^), a likely result of their demographic history. By contrast, no significant correlation was found between the pair-wise *Q*_*ST*_ values for virus accumulation or *SW*_*i*_*/SW*_*m*_, for any CMV isolate, and their geographic distances (*r*≤0.24, *P*≥0.258). Therefore, factors other than demography contribute to the population differentiation patterns observed for resistance and tolerance to CMV.

## Discussion

Host-pathogen coevolution determines the dynamics and genetics of infectious disease, and may shape the genetic structure of host and pathogen populations [1]. Understanding this topic, central in pathology and evolutionary biology, requires knowledge on the genetics of defence and pathogenicity, and the dynamics of their change in populations [1]. The abundance of theoretical analyses of host-pathogen coevolution (e.g., [1,56,57]) is not matched by a similar amount of empirical and experimental studies. While there is abundant information compatible with host-pathogen coevolution in plant systems (e.g. [58]), it mostly derives from crops, in which the genetic composition of the host plant is manipulated by humans. Studies from wild systems, in which the genetic composition of host and pathogen populations may be determined by reciprocal selection, are much scarcer, particularly for plant-virus interactions [5,59]. To address this question we challenged *A*. *thaliana* individuals collected from a high number of local populations with different CMV isolates. We chose to inoculate plants mechanically rather than by aphid transmission, which is the natural means of horizontal transmission [39]. Mechanical inoculation ensures a high rate of infection and minimises inoculum dose effects on virus accumulation, as opposed to aphid transmission, which is highly inefficient for CMV [39, 40]. Moreover, there is no information on the aphid species that transmit CMV in *A*. *thaliana* populations in central Spain. Also, *A*. *thaliana* genotypes were assayed under common controlled conditions, which are not necessarily the same as in the field.

The challenge with different CMV strains of 185 individuals collected in 76 *A*. *thaliana* local populations from the Iberian Peninsula, showed large differences in quantitative resistance, as estimated from the level of virus multiplication in infected plants. Similarly, large differences were found for tolerance to CMV, estimated as the effect of infection on host progeny production. A large part of the variation of resistance and tolerance was explained by the virus isolate, in agreement with previous results showing that CMV isolates vary largely in multiplication rate and virulence in *A*. *thaliana* [41,47,48,53]. Variation for resistance and tolerance occurred at all analysed spatial scales: among individuals from a local population, among local populations, and within the whole Iberian Peninsula region. These conclusions held for assays conducted in different environments, a result to be underscored, as resistance and tolerance of *A*. *thaliana* to CMV can be modulated by the abiotic environment [14]. The observed variation in resistance and tolerance had a significant genetic component, as they showed medium to high heritability values (0.23–0.81 for resistance, and 0.10–0.70 for tolerance) depending on the spatial scale of the analysis and the isolate of CMV. Thus, our results show genetic variation for presumably defence traits in the host population, a condition for host-pathogen coevolution. Regardless of spatial scales, our data also show that resistance and tolerance significantly depend on the interactions between virus genotype and host genotype, another condition for host-pathogen coevolution.

It has been reported that *A*. *thaliana* genotypes showing high tolerance to CMV have a long life span, and that tolerance is, at least in part, the result of resource re-allocation from vegetative growth to reproduction, which is more efficient in long-lived genotypes [14,47,48,52]. It also has been shown that these life history and phenological traits have evolved as (direct or indirect) responses to climatic conditions [33,49,50,60]. Accordingly, the analysis of 76 *A*. *thaliana* genotypes from different Iberian populations showed that resource reallocation upon infection depended on the allometric ratio *(SW+IW)/RW*, and the effect of CMV infection on vegetative growth correlated positively with *LP* and *RW* of mock-inoculated plants. Life span, vegetative growth and seed production in non-infected plants were correlated with climatic variables. In contrast, the genetic variation for CMV multiplication in the infected host, and for the effect of CMV infection on seed production, was unrelated to those climatic factors. Therefore, the CMV defence traits have evolved, at least partly, independently from those other adaptive traits which have evolved in response to climate. Accordingly, the evolution of defence traits is not the result of *A*. *thaliana* responses to climate. These differential evolutionary histories strongly suggest that these traits are true resistance and tolerance defence responses that may have evolved in response to CMV infection (see below). This conclusion also agrees with the fact that resistance and tolerance are virus-specific traits of *A*. *thaliana*, and not unspecific responses to the stress of virus infection [52]. Thus, our study shows substantial genetic variation for resistance and tolerance to CMV within and between populations of *A*. *thaliana*. Genetic variation within or/and between populations for resistance to a variety of pathogens has been reported for a limited number of wild plants, including *Amphicarpaea bracteata*, *Eucalyptus globulus*, *Podophyllum peltatum*, *Linum marginale*, *Silene latifolia*, *Phaseolus vulgaris*, *Plantago laceolata* or *A*. *thaliana* [61–78]. Analyses of the variation for plant tolerance to pathogens are much rarer [47,75,79], and none of them has analysed within population variation. All these studies refer to resistance or tolerance to fungi, oomycetes or bacteria, and the only report we are aware of on variation for resistance to a virus, is our previous analysis in the *A*. *thaliana*-CMV system [43], which involved a much more limited sample of host populations.

The analyses in this study also showed that resistance and tolerance display different evolutionary histories than neutral genetic variation, since *Q*_*ST*_ values for resistance and tolerance are lower than *F*_*ST*_ values. Accordingly, resistance and tolerance are traits likely under uniform selection, i.e., a selection which favours a higher diversity of traits within than among populations. This conclusion is supported by results for two CMV isolates and in different environmental conditions. Also, neutral genetic differentiation follows a pattern of isolation by distance, which is not detected for the genetic differentiation for resistance or tolerance. By contrast, similar analyses have previously suggested diversifying selection on the genetic variation for quantitative traits such as flowering time, leaf number, specific leaf area or leaf succulence in *A*. *thaliana* [32,80,81]. The most parsimonious explanation for uniform selection on CMV defence traits is that CMV infection plays a role as selective factor. Although we cannot discard that the analysed defence traits may have evolved in response to selection from other pathogens or pests, several arguments make a strong case for selection pressure due to CMV infection: i) the high prevalence of CMV (up to 80%) in wild *A*. *thaliana* populations in the Iberian peninsula [43,44]; ii) the fact that the analysed defence traits are virus-specific in *A*. *thaliana* [52]; and iii) the lack of correlation between CMV multiplication and virulence [47], virus multiplication being highly dependent on virus genotype and environment [14,47]. Selection for resistance to pathogens has been best documented in populations of *Linum marginale* in response to the fungus *Melampsora lini*, of *Plantago lanceolata* in response to the fungus *Podosphaera plantaginis*, or of *A*. *thaliana* in response to the oomycete *Hyaloperonospora arabidopsidis* or the bacterium *Pseudomonas syringae* [37,68,69,72–74,78,82,83]. Our results extend these observations to plant-virus interactions. If defence in *A*. *thaliana* against CMV is under selection, a corollary is that CMV is a virulent pathogen of this plant under natural conditions, a relevant conclusion that contributes significantly to understanding plant-virus interactions.

The observed pattern of genetic diversity for resistance and tolerance to CMV, higher within than between populations, can be explained by the features of the pathosystem. CMV is ubiquitous in Iberia [45,84] and has been found in all monitored wild populations of *A*. *thaliana*, prevalence differing among populations and years [43]. Also, our present and past results [47,48] show that CMV isolates differ in virulence to *A*. *thaliana*, and that virulence is modulated by environmental factors as diverse as temperature, light intensity or host plant density [14,53].Variation in the genetic composition of CMV populations would also result in variation for CMV infection-associated selection, as the outcome of the interaction depends on the *A*. *thaliana* and CMV genotypes involved. These factors would explain the maintenance of genetic variation in defence traits within host populations and the limited differentiation among populations. Furthermore, this explanation suggests that resistance and tolerance to CMV involve fitness penalties for *A*. *thaliana*, which would hinder fixation of resistance/tolerance alleles and would contribute to the maintenance of defence polymorphisms within populations [85]. We have not found evidence for such costs under the assayed conditions, as neither resistance nor tolerance were negatively correlated with viable seed production of mock-inoculated controls. However, fitness costs might not be detectable under our experimental conditions, and/or might be unveiled under the less favourable environment of the field.

Another interesting result is that resistance and tolerance to CMV co-occur in wild *A*. *thaliana* populations. Theory predicts that resources being limited, hosts would not invest in both resistance and tolerance, which would be mutually exclusive defences. The conditions that should favour the evolution of resistance or tolerance have been much modelled, and a negative correlation between both traits across host genotypes is expected [4,86,87]. We found no correlation between resistance and tolerance in any experiment, indicating that they evolve as independent traits. It has been proposed that resistance and tolerance could coexist if costs and benefits of each defence were different and non-additive [88–91]. Models also propose that tolerance alleles should become fixed in host populations, which would not be polymorphic for this trait under most assumptions [91–93]. A report on the tolerance to CMV in *Mimulus guttatus* conforms to these predictions, as tolerance had no costs, but showed little genetic variation [94,95]. On the contrary, our results do not agree with model predictions, as we found large genetic variation for tolerance and evidence for coexistence of resistance and tolerance.

In conclusion, this work shows that in *A*. *thaliana* there is genetic variation, within and among populations, for defences to CMV that result in lower virus multiplication or in lower impact of infection on the plant fitness. Genetic variation for defence to CMV is not associated with variation for climatic factors, in contrast to variation for other adaptive life-history traits of *A*. *thaliana*. In addition, we found evidence that these two defence traits are under uniform selection. The results of this study are compatible with CMV infection acting as a selection pressure for defence on populations of *A*. *thaliana* and, hence, we propose that CMV infection likely reduces the host fitness under the field conditions of the analysed wild *A*. *thaliana* populations, although field experiments would be required to prove this fact. The results presented here also show that some of the conditions for coevolution are met in the system *A*. *thaliana*-CMV, but more work on the virus side is necessary to prove if coevolution occurs. These results raise two challenging questions: what are the mechanisms that maintain polymorphisms for resistance and tolerance within *A*. *thaliana* populations, and what is the negative impact of CMV infection on the host in nature and how does such an impact vary according to field conditions.

## Materials and methods

### Plant material and environmental data

Two different sets of *A*. *thaliana* samples from the Iberian Peninsula were analysed. First, 76 accessions or wild genotypes collected from different populations were selected to cover the genetic and environmental diversity of the species in that region [30,49] (Fig 1 and S1 Table). This collection spanned 800 x 700 km, populations being spaced in the average 384.9±3.7 (20.2–1,038.1 km). Altitudes ranged from 123 to 1,670 m above sea level. Each sample was genetically different based on previous SNP genotyping and genome sequences [51,96]. Second, ten individuals plants (individuals) randomly sampled from 12 of these populations were selected for intra and inter-population analyses (Fig 1 and S1 Table). Samples from eight of these populations have been previously genotyped for 250 genome-wide SNPs that were segregating in these populations [30,32]. For the remaining four populations (Bis, Mer, Moc, Pob) 10 individuals/population were genotyped in this study for the same set of SNPs.

The climatic information from the locations of *A*. *thaliana* populations was obtained from the digital climatic atlas of the Iberian Peninsula at 1 km^2^ resolution [50,97]. Thirty-three variables were used, related to temperature, precipitation and solar radiation (S3 Table). In addition, 19 bioclimatic variables derived by combination of annual trends, seasonality and extreme conditions were also included (www.worldclim.org). Altitude was also analysed as a proxy for climate. Annual mean temperature of the populations ranged 6.1–17.4°C (12.5±0.3) and annual precipitation ranged 405.7–1695.8 mm (753.8±33.9) (S8 Table).

All accessions or individuals used in this study were propagated by selfing during two generations by the single seed descent procedure, in a glasshouse supplemented with lamps to provide a long-day photoperiod. This allowed reducing residual heterozygosity that might contain some wild individuals but also removing any potential maternal and grand-mother effects. Seeds were stratified (darkness, 4ºC) for 7 days before germination at 25/20ºC day/night, 16 h light. Ten day-old seedlings were transferred to 4ºC, 8 h light, for vernalisation during 4 or 8 weeks, depending on the experiment. After vernalisation, plants were transplanted to 0.43 L pots and returned to the greenhouse, where they were kept (25/20ºC day/night, 16 h light) until the end of the experiment.

### Virus isolates and inoculations

Three subgroup IA CMV isolates were used, Fny-CMV, Cdc-CMV and Lro-CMV, which differ in the sequence of their genomic RNAs in about 1% of positions. Fny-CMV is a well-characterized reference isolate [98]. Cdc-CMV and Lro-CMV were isolated from field-infected *A*. *thaliana* plants of the Cdc and Cho populations, respectively, in 2008 and 2011, Cdc-CMV was named At-CMV in [43]. Isolates were multiplied in *Nicotiana clevelandii*, Fny-CMV from transcripts of cDNA clones and Cdc-CMV and Lro-CMV from biological clones derived from local lesions in *Chenopodium quinoa*. Virions were purified as in [99]. *A*. *thaliana* plants were mechanically inoculated at the five-leaf stage (stage 1.05, [100]) with 15 μl of sap from infected *N*. *clevelandii* leaves in 0.01 M phosphate buffer pH 7.0, 0.2% sodium diethyldithiocarbamate. Fifteen μl of buffer were applied to mock-inoculated controls. The (unkown) virus concentration in leaf sap ensured infection of 100% of inoculated plants. Each treatment (virus-inoculated or buffer mock-inoculated) involved at least five replicated plants from each original sample, that is at least five plants derived from the same genotype or individual. All plants in each experiment were grown in a completely randomized design.

### Quantification of CMV multiplication

Virus multiplication in plants was estimated from virus RNA accumulation as described in Pagán *et al*., (2014) [41]. Briefly, at fifteen days post-inoculation 0.01 g fresh weight (fwt) of leaf tissue was harvested from four different systemically infected leaves. Nucleic acids were extracted from the pooled leaf tissue using TRI-reagent (Sigma-Aldrich, St Louis, MO, USA). Virus RNA was then quantified by dot-blot hybridization with ^32^P-labelled RNA probes complementary to nucleotides 1933–2215 of Fny-CMV RNA3 (GeneBank Acc. No. D10538). In each blot, internal standards for Fny-CMV, Cdc-CMV or Lro-CMV RNA were included as a two-fold dilution series (1–0.001 μg) of purified virion RNA in nucleic acid extracts from non-inoculated plants. Mock-inoculated samples served as negative controls. Nucleic acid extracts were blotted at different dilutions to ensure that hybridization signal was on the linear portion of the RNA concentration-hybridization curve. As loading controls, parallel membranes were hybridized with a cDNA probe of β-tubulin chain 2 (TUB2) mRNA of *A*. *thaliana* (1086–1568 nt, GeneBank Acc. No. NM_125664.4).

### Quantification of life-history traits and tolerance to CMV

Rosette weight was used to estimate vegetative growth effort, inflorescence plus seed weight to estimate total reproductive effort, and seed weight to estimate progeny production [101]. Previous work has shown that CMV infection does not affect seed viability, nor the weight of individual seeds, in a broad range of *A*. *thaliana* genotypes [14,47,52]. Plants were harvested at complete senescence and dry weight of rosettes (rosette weight, *RW*), inflorescence structures without seeds (inflorescence weight, *IW*) and seeds (seed weight, *SW*) were measured separately (g). Two phenological parameters of *A*. *thaliana* life cycle were quantified: Growth period (*GP*) and life-span (*LP*) were measured as the time (days) between planting seedlings in soil and opening of the first flower (*GP*), or complete senescence (*LP*). Tolerance was measured by the effect of virus infection on progeny production: *SW*_*i*_*/SW*_*m*_, where *i* and *m* denote infected and mock inoculated plants, respectively [48].

### Genetic analyses

Broad sense heritability of each trait was estimated as *h*^*2*^_*b*_ *= V*_*G*_*/(V*_*G*_*+V*_*E*_*)*, where *V*_*G*_ is the among-genotypes or among-populations variance component and *V*_*E*_ is the residual variance. Variance components were determined using the REML method [102] of SPSS 20 package (SPSS Inc., Chicago, USA).

Genetic differentiation between populations for quantitative traits was measured by *Q*_*ST*_ values [103], estimated as *V*_*B*_*/(V*_*B*_*+V*_*W*_*)* [104,105], where *V*_*B*_ is the between-population variance and *V*_*W*_ is the within-population variance. *V*_*B*_ and *V*_*W*_ were estimated by the REML method from a nested analysis of variance performed using population and individual or genotype (nested within populations) as random factors. The 95% confidence intervals (95%CI) for *Q*_*ST*_ values were estimated as $P\left[\frac{S^{2}(n-1)}{\chi_{n-1}^{2}}\leq \sigma^{2}\leq \frac{S^{2}(n-1)}{\chi_{n-1}^{2}}\right]=0.95$ [106]. Genetic differentiation for neutral markers was estimated as *F*_*ST*_ [107] using the analysis of molecular variance (AMOVA) as implemented in ARLEQUIN v3.5.1.2 [108]. AMOVA were performed using multilocus genotypes for 250 segregating SNPs [30,32] and their significances were estimated from 1,000 permutations.

The relationships between Euclidean geographical distance and *F*_*ST*_ or *Q*_*ST*_ values among population pairs were determined by Mantel correlation test using PASSaGE v.2 [109] with 1,000 permutations. Genetic distances between individuals were calculated as the proportion of allelic differences over the total number of alleles in the corresponding set of polymorphic loci, using GGT v. 2.0 [110].

### Statistical analyses

Differences in *RW*, *IW*, *SW*, *GP*, *LP*, virus accumulation or tolerance to CMV, according to host individual/genotype and virus isolate, were analysed by general linear models (GLM) considering host individual/genotype as a random factor, and virus isolate as a fixed factor. Differences in *RW*, *IW*, *SW*, *GP*, *LP*, virus accumulation or tolerance to CMV according to population, host individual/genotype, and virus isolate, were analysed by GLM considering isolate as a fixed factor, and population and individual/genotype nested to population, as random factors. Relationships between values of different traits were tested using Spearman’s correlation test. GLMs and Spearman’s correlation tests were performed using SPSS 20 software package.

Spatial autocorrelation patterns of environmental variables, life-history traits, virus accumulation and tolerance to virus, were analysed using correlograms [111] generated with PASSaGE v.2. For each variable, Moran’s *I* autocorrelation coefficients [112] were calculated and their significance tested from 1,000 permutations. Correlation between pairs of environmental variables, between pairs of different traits and between environmental variable and different traits were tested with Dutilleul’s modified t-test using SAM v.4 [113,114]. Simultaneous autoregressive models (SAR) [115] were performed to test the relationship between environmental variables and different traits using SAM v.4. Bonferroni correction was applied for multiple comparisons.

The relationships between environmental variables and life-history or defence traits were analysed by partial Mantel tests controlling for the location of populations given by the geographic distance matrix using PASSaGE v.2. For that, matrices of euclidean distances were derived for each environmental variable and phenotypic trait and significance was evaluated from 1,000 permutations.

## Supporting information

S1 Table Geographic origin and location of Iberian *A. thaliana* populations. (XLSX)

S2 Table GLM analysis of values of life history traits of mock-inoculated *A. thaliana* plants, of tolerance to CMV, and of virus accumulation, using "host genotype" as a random factor. For each trait, heritability (*h*^*2*^_*b*_), mean, minimum and maximum values, are shown.
(XLSX)

S3 Table Values of climatic variables for 76 Iberian *A. thaliana* populations, and for the phenotypic traits of representative individuals. (XLSX)

S4 Table Coefficients of the correlation/regression between values of mock-inoculated *A. thaliana* life-history or defence to CMV traits, and those of the environmental variables of the corresponding population obtained by SARs, Correlation with Dutilleul´s correction, and Mantel test. A threshold significance value (α = 0.0014) was set after applying Bonferroni's correction (1– [1– a] 1/n) for multiple comparisons.
(XLSX)

S5 Table Mean, minimum and maximum values of resistance and tolerance to CMV of *A. thaliana* individuals inoculated after a four-week vernalisation period. Mean, minimum and maximum values, heritability and *Q*_*ST*_ of resistance and tolerance to CMV for twelve *A*. *thaliana* populations are also shown.
(XLSX)

S6 Table Mean values of resistance and tolerance to CMV for *A. thaliana* individuals inoculated after an eight-week vernalisation period. (XLSX)

S7 Table Distribution of genotypes (H) and individuals (lower case three letter codes) in 12 analysed *A. thaliana* populations. (XLSX)

S8 Table Values (mean and range of variation) of the environmental variables used in the study. Values are the averages of 76 population.
(XLSX)
